# Efficacy and Durability of Two Desensitizing Agents on Dentinal Tubule Occlusion: An In Vitro Study

**DOI:** 10.7759/cureus.74189

**Published:** 2024-11-21

**Authors:** Mounika Narra, Lavanya Anumula, Suneel Kumar Chinni, Kiranmayi Govula, Swapna Sannapureddy, Karthik Nagella

**Affiliations:** 1 Conservative Dentistry and Endodontics, Narayana Dental College and Hospital, Nellore, IND; 2 Conservative Dentistry and Endodontics, Narayana Dental College and Hospital, Nelllore, IND

**Keywords:** dentin sensitivity, gluma, hydrodynamic, quality of life, silver fluoride, tooth brushing

## Abstract

Background: Dentin hypersensitivity (DH) is a common condition caused by exposed dentinal tubules, often requiring treatment with desensitizing agents. This in vitro study conducted at Narayana Dental College and Hospital (Nellore, AP, IND) between January 2022 and March 2022, aimed to evaluate the effectiveness of two desensitizing agents, SDI Riva Star (SDI Ltd., Bayswater, VIC, AUS) and Gluma Desensitizer (Kulzer, Hanau, DEU) in occluding dentinal tubules and their long-term durability following simulated brushing.

Materials and methods: Fifty maxillary premolar specimens were sectioned and treated with 17% ethylenediamine tetraacetic acid (EDTA) to expose dentinal tubules. They were divided into three groups: (I) a control group, (II) SDI Riva Star, and (III) Gluma Desensitizer. Tubule occlusion was assessed using scanning electron microscopy (SEM) immediately after application and after one month of brushing.

Results: Both test agents occluded the dentinal tubules effectively (SEM score 1), with no significant difference between SDI Riva Star and Gluma Desensitizer immediately post-application. However, after 30 days of brushing, SDI Riva Star demonstrated superior durability, with significantly better occlusion scores than Gluma Desensitizer (p<0.01).

Conclusion: Both SDI Riva Star and Gluma Desensitizer were effective in occluding dentinal tubules, but SDI Riva Star showed greater durability over time, making it a more effective option for long-term desensitization.

## Introduction

Dentin hypersensitivity (DH) is a prevalent clinical condition characterized by short, sharp pain that arises from exposed dentin in response to various stimuli, such as thermal, tactile, osmotic, or chemical changes [1]. These stimuli typically affect the dentin when the protective enamel or cementum covering is lost, exposing the dentinal tubules to the oral environment. The exposed dentin becomes vulnerable to external stimuli, causing discomfort or pain. This condition affects daily life, reducing the patient’s quality of life by limiting their ability to enjoy certain foods and drinks.

The pathophysiology of DH is linked to the structure of dentin, a mineralized tissue that contains fluid-filled tubules extending from the outer surface of the dentin to the pulp. These dentinal tubules are lined with odontoblastic processes, and when exposed, the movement of fluid within the tubules stimulates nerve fibres in the pulp, causing sensitive dentin. According to the hydrodynamic theory proposed by Brannström, this fluid movement, caused by external stimuli, is the primary cause of pain in DH [2]. The condition is also associated with factors such as erosion, abrasion, and gingival recession that contribute to the exposure of dentinal tubules.

Various treatment approaches have been developed to address DH, with a focus on either occluding the exposed dentinal tubules or desensitizing the nerve fibres (A-delta fibres) to reduce sensitivity. Common desensitizing treatments include potassium-based agents, which act by blocking nerve activity and tubule-occluding agents, which seal the dentinal tubules to prevent fluid movement [3]. The occlusion of dentinal tubules remains one of the most effective strategies for treating DH. Desensitizing agents commonly used in clinical practice include fluoride varnishes, oxalates, self-etch, and total-etch bonding agents, as well as composite and resin-modified glass ionomer cement. These materials work by forming a physical barrier over the tubules or precipitating within the tubules to block fluid movement.

The effectiveness of these agents, however, depends not only on their ability to occlude the tubules but also on the durability of this occlusion over time. Daily oral hygiene practices, such as tooth brushing, can erode the protective layer formed by desensitizing agents, reducing their long-term efficacy [4]. Therefore, an ideal desensitizing agent should not only provide immediate relief but also maintain its protective effects after prolonged exposure to mechanical forces such as brushing.

In this context, the current study focuses on evaluating the efficacy of two desensitizing agents, silver diamine fluoride (SDF) SDI Riva Star (SDI Ltd., Bayswater, VIC, AUS) and Gluma Desensitizer (Kulzer, Hanau, DEU), in occluding dentinal tubules and assessing the durability of their effects after one month of simulated brushing. Silver diamine fluoride SDI Riva Star is a relatively newer agent containing silver fluoride and potassium iodide, known to form low-solubility precipitates within dentinal tubules. This precipitate acts as a physical barrier, preventing fluid movement and reducing sensitivity. Gluma Desensitizer, on the other hand, is a widely used desensitizing agent that contains 5% glutaraldehyde and 35% hydroxyethyl methacrylate (HEMA) [5]. Glutaraldehyde is believed to work by coagulating proteins within the dentinal fluid, while HEMA forms a resin that occludes the tubules [6].

Previous studies have shown both agents to be effective in the immediate reduction of DH symptoms by occluding dentinal tubules [7,8]. However, there is limited data on their comparative performance over time, especially in their ability to withstand the mechanical stresses of toothbrushing. Therefore, this study aims to compare the immediate occlusion of dentinal tubules by SDI Riva Star and Gluma Desensitizer, as well as their durability after 30 days of simulated brushing. The null hypothesis (H₀) for this study is that there is no significant difference between SDI Riva Star and Gluma Desensitizer in terms of their ability to occlude dentinal tubules and maintain occlusion over time, even after 30 days of brushing.

## Materials and methods

Study design and sample selection

This in vitro study was conducted at Narayana Dental College and Hospital, Nellore, AP, IND, between January 2022 and March 2022, following approval from the Institutional Ethics Committee (approval no. IEC/NDCH/2021/P-10). The study adhered to the ethical guidelines for research on extracted human teeth. A total of 25 freshly extracted, non-carious human maxillary first premolar teeth were selected for the study. Teeth with decay, fractures, or malformations were excluded. The specimens were stored in saline at room temperature immediately after extraction to prevent dehydration.

Specimen preparation and experimental groups

Class V cavities measuring 2 mm in depth and 3 mm in width were prepared on the buccal and lingual surfaces of the teeth using a 245 carbide bur in an air-rotor handpiece. Each tooth was then sectioned mesiodistally with a diamond wheel disc to obtain 50 specimens, including 25 buccal and 25 lingual surfaces. The dentin surfaces were polished with abrasive paper to ensure uniform exposure, followed by treatment with 17% ethylenediamine tetraacetic acid (EDTA) for 40 minutes [9] to open the dentinal tubules completely. The 50 specimens were randomly divided into five distinct groups, categorized by the treatment applied and the timing of testing (Table 1).

**Table 1:** Overview of the experimental groups, treatments, and testing conditions

Groups	Treatment	Timing of testing
Group I (control)	No treatment applied	Not applicable
Group II	SDI Riva Star	Group IIa: Tested immediately after application for tubule occlusion; Group IIb: Tested for durability after one month of brushing
Group III	Gluma Desensitizer	Group IIIa: Tested immediately after application for tubule occlusion; Group IIIb: Tested for durability after one month of brushing

Application of desensitizing agents 

Silver diamine fluoride SDI Riva Star was applied in two steps as per the manufacturer’s instructions. The first step involved applying silver fluoride to the dentin surface using a disposable brush, followed by the application of potassium iodide until the surface turned clear. Gluma Desensitizer was applied with a disposable brush to the dentin surface and left in place for 36 seconds before rinsing with water.

Simulated brushing for durability testing

Specimens in group IIb and group IIIb were subjected to simulated brushing for two minutes twice daily for 30 days using a brushing simulator (SD Mechatronik, Stuttgart, DEU). The brushing was performed with a load of 250 g and an oscillation of 6800 strokes per minute. In between brushing sessions, the specimens were stored in artificial saliva (Wet Mouth, ICPA Health Products Ltd., Mumbai, MH, IND) to simulate the oral environment.

Evaluation of dentinal tubule occlusion and statistical analysis

The specimens were mounted on metal stubs, air-dried, and sputter-coated with a 25 nm layer of gold for scanning electron microscopy (SEM) analysis (JSM-IT800, JEOL Ltd., Tokyo, JPN), at a magnification of 3000x, to assess dentinal tubule occlusion. The tubule occlusion was manually scored by an independent evaluator who was blinded to the different groups involved in the study. The scoring was performed using the West et al. scoring system (Table 2) [10]. The results were analysed using SEM images and statistical tests via SPSS Statistics, version 21 (IBM Corp., Armonk, NY, USA), including the Kruskal-Wallis one-way analysis of variance and the Mann-Whitney U test for intergroup comparisons. A p-value of <0.05 was considered statistically significant.

**Table 2:** Scoring system for dentinal tubule occlusion based on SEM analysis SEM: Scanning electron microscopy

Score	Tubule occlusion description
1	100% of tubules occluded
2	50% to <100% of tubules occluded
3	25% to 50% of tubules occluded
4	<25% of tubules occluded
5	0% of tubules occluded

## Results

Tubule occlusion immediately after application

**Figure 1:**
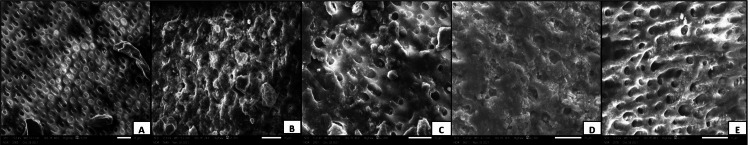
The SEM images illustrating dentinal tubule occlusion across different experimental groups A: Control group showing fully open, untreated tubules; B: Group IIa (SDI Riva Star) showing occluded tubules immediately after application; C:  Group IIIa (Gluma Desensitizer) showing occluded tubules immediately after application; D: Group IIb (SDI Riva Star) after 30 days of simulated brushing, demonstrating minimal reopening of tubules; E: Group IIIb (Gluma Desensitizer) after 30 days of simulated brushing, showing partial reopening of tubules SEM: Scanning electron microscopy

The SEM analysis of specimens treated with SDI Riva Star and Gluma Desensitizer demonstrated significant dentinal tubule occlusion immediately after application. Both agents showed near-complete occlusion (SEM score 1), with no significant difference between them. In contrast, the control group, which did not receive any desensitizing treatment, exhibited fully open dentinal tubules, with SEM scores ranging from 4 to 5 (Figure 1).

Table 3 provides a detailed intergroup comparison of the immediate occlusion scores between the control, SDI Riva Star, and Gluma Desensitizer groups. The Kruskal-Wallis one-way analysis of variance showed a highly significant difference between the control group and the two test groups (p < 0.001). Post-hoc pairwise comparisons (Table 4) further revealed that the mean difference between the control group and SDI Riva Star (IIa) was significant (mean difference = 3.60000, p = 0.000), as well as between the control group and Gluma Desensitizer (IIIa) (mean difference = 3.50000, p = 0.000). However, the difference between SDI Riva Star (IIa) and Gluma Desensitizer (IIIa) was not significant (mean difference = -0.10000, p = 0.542), indicating that both desensitizing agents had similar immediate effects on tubule occlusion.

**Table 3:** Intergroup comparison of desensitizing agents among the control group, SDI Riva Star group, and Gluma Desensitizer group using the Kruskal-Wallis one analysis of variance (p ≤0.05 significant)

Group	n	Min	Max	Mean	SD	p-value	Result
Control	10	4	5	4.70	0.48	<0.001	Significant
SDI Riva Star (II a)	10	1	2	1.10	0.32		
Gluma Desensitizer (III a)	10	1	2	1.20	0.42		

**Table 4:** Pairwise comparison using the Mann-Whitney U test (p<0.05* significant)

Groups		Mean difference	p-value	Result
Control	SDI (IIa)	3.60000^*^	0.000	Significant
	Gluma (IIIa)	3.50000^*^	0.000	Significant
SDI Riva Star (IIa)	Gluma (IIIa)	-0.10000	0.542	Not significant

Durability of Tubule Occlusion After 30 Days of Brushing (Figure 1): After 30 days of simulated brushing, SEM analysis indicated a reduction in tubule occlusion for both desensitizing agents; however, SDF Rivastar demonstrated greater tubule occlusion retention compared to Gluma.

Table 5 presents the intergroup comparison of brushing results between SDF Rivastar and Gluma, after 30 days of brushing. The Mann-Whitney U test revealed a statistically significant difference in tubule occlusion between the two desensitizing agents. (p = 0.010).

**Table 5:** Intragroup comparison of tubule occlusion for SDF Rivastar and Gluma after thirty days of brushing using Mann-Whitney U test (P ≤0.05 – Significant).

Group	Min	Max	Mean	SD	P value	Result
SDF Rivastar (IIb)	1	2	1.60	0.52	0.010	Significant
Gluma (III b)	2	3	2.30	0.48		

## Discussion

The findings from this in vitro study highlight the immediate and durable effectiveness of SDI Riva Star and Gluma Desensitizer as desensitizing agents. Both agents successfully occluded dentinal tubules immediately post-application. However, their performance diverged after 30 days of simulated brushing, where SDI Riva Star demonstrated superior retention of tubule occlusion compared to Gluma Desensitizer.

Scanning electron microscopy was selected due to its capacity for high-resolution, three-dimensional, non-destructive, and objective imaging. This technique has been consistently utilized in prior research to evaluate dentinal tubule occlusion [11-14]. Chen et al. [15] used SEM to assess the durability of desensitizing agents after an acid challenge, simulating oral conditions from acidic foods and beverages. This helps to evaluate the effectiveness of the agents and their resistance to acid, ensuring lasting relief from DH under daily dietary stresses.

The initial results of this study are consistent with the findings from various previous investigations. Gluma Desensitizer, a well-established desensitizing agent containing glutaraldehyde and HEMA, has been recognized for its efficacy in sealing dentinal tubules. Dundar et al. demonstrated that desensitizing agents like Gluma Desensitizer significantly reduce fluid conductance by occluding tubules, which aligns with the immediate occlusion observed in our study [16]. Similarly, Mushtaq et al. reported that Gluma Desensitizer showed excellent tubule occlusion when assessed under SEM immediately after application ​(group IIIa) [9].

Silver diamine fluoride SDI Riva Star, a relatively newer agent combining silver fluoride and potassium iodide, blocks the microscopic tubules that make up dentin. A low-solubility precipitate (silver iodide) occludes dentinal tubules, giving instant relief. The work by Kiesow et al. highlighted that silver fluoride-based desensitizers provide strong occlusion through the formation of insoluble silver complexes within the tubules, effectively blocking fluid movement [17]. This mechanism likely explains the immediate success of SDI Riva Star in our study, where SEM analysis revealed complete or near-complete occlusion of tubules (group IIa).

The key difference between SDI Riva Star (II b) and Gluma Desensitizer (III b) became evident after 30 days of simulated brushing. Our results indicate that SDI Riva Star maintained significantly more tubule occlusion compared to Gluma Desensitizer, which experienced partial reopening of tubules. Abuzinadah et al. found that silver-based agents such as SDI Riva Star not only effectively block tubules but also resist mechanical stress, such as brushing [18]. The durability of silver fluoride is likely due to the formation of a stable precipitate within the tubules, which is less soluble and more resistant to wear [19].

On the other hand, the results for Gluma Desensitizer (III b) align with earlier findings by Kumar et al. [20], who reported that while Gluma Desensitizer provides effective immediate relief from DH, its efficacy diminishes over time due to the breakdown of the resin matrix under mechanical stress. The hydrophilic nature of HEMA in Gluma Desensitizer may contribute to this loss of occlusion, as it could allow for some fluid penetration and degradation of the occlusive barrier, particularly under the abrasive forces of brushing.

The differences in the long-term performance of these agents have important implications for clinical practice. Dentists often seek desensitizing treatments that not only provide immediate relief but also offer sustained protection against stimuli. Given its superior durability, SDI Riva Star may be a more suitable choice for patients with severe or chronic DH, particularly those who perform rigorous oral hygiene practices. The longevity of its occlusive effect can minimize the need for frequent reapplications, which is a common drawback of many desensitizing agents (including Gluma Desensitizer). Gluma Desensitizer, while still effective, may be more appropriate for patients with mild to moderate hypersensitivity or those who are willing to undergo periodic reapplications to maintain optimal results.

Class V cavities were chosen in this study to replicate clinical conditions where dentin exposure is common, particularly in the cervical third of the tooth. This region is often affected by non-carious cervical lesions, gingival recession, or root surface exposure, making it a relevant area for assessing the effectiveness of desensitizing agents. The anatomy of dentinal tubules in the cervical third is unique, as the tubules are generally larger in diameter and more numerous compared to other regions, increasing fluid movement and sensitivity. This makes the cervical third a particularly challenging area for effective desensitization, highlighting its importance in our study.

Artificial saliva was used to simulate the oral environment, maintaining hydration and mimicking conditions that influence dentin and the action of desensitizing agents. The pH of the artificial saliva was maintained at around 6.8 to 7.0, closely resembling the neutral pH of natural saliva, ensuring that the test conditions do not artificially favour or inhibit the performance of the desensitizing agents, allowing for a more accurate representation of their durability and effectiveness in an oral setting.

The comparison between SDI Riva Star and Gluma Desensitizer in this study mirrors the findings of similar comparative studies. Makkar et al. [21] evaluated the tensile bond strength of various desensitizing agents and found that resin-based systems like Gluma Desensitizer performed well initially but were outperformed by silver fluoride-based agents in terms of long-term durability. Furthermore, Pamir et al. [22] demonstrated that desensitizing agents based on silver fluoride showed better resistance to mechanical forces and maintained occlusion for extended periods, supporting the outcomes observed in our study.

While this study focused on SDI Riva Star and Gluma Desensitizer, other desensitizing agents, such as bioactive glass and potassium oxalate, have also been shown to provide effective relief from DH. For instance, Erdemir et al. [23] explored the use of sealants like Seal & Protect (Dentsply Sirona Inc., Charlotte, NC, USA), which showed prolonged occlusion of dentinal tubules but faced challenges similar to Gluma Desensitizer in terms of long-term durability. Future research could expand on these findings by comparing the performance of SDI Riva Star with other advanced materials such as bioactive glasses or calcium phosphates, which have shown promise in promoting remineralization and tubule sealing.

Limitations

Since this is an in vitro study, it's important to note that the experimental conditions, like simulated brushing, may not completely capture all the complexities of the oral environment. This could be a major limitation. Factors such as saliva, dietary habits, and microbial activity could impact the long-term effectiveness of these desensitizing agents in a real-life setting.

## Conclusions

This study demonstrated that while SDI Riva Star and Gluma Desensitizer effectively occluded dentinal tubules immediately after application, the former showed superior durability in maintaining occlusion after 30 days of simulated brushing. These findings support the use of SDI Riva Star as a more durable option for managing DH, particularly in patients requiring long-term relief. Further clinical research is needed to corroborate these results and explore new approaches to enhance the longevity and effectiveness of desensitizing treatments.
